# Native and oxidised lipoproteins negatively regulate the serum amyloid A‐induced NLRP3 inflammasome activation in human macrophages

**DOI:** 10.1002/cti2.1323

**Published:** 2021-08-03

**Authors:** Katariina Nurmi, Katri Niemi, Ilona Kareinen, Kristiina Silventoinen, Martina B Lorey, Yan Chen, Vesa‐Petteri Kouri, Jukka Parantainen, Timo Juutilainen, Katariina Öörni, Petri T Kovanen, Dan Nordström, Sampsa Matikainen, Kari K Eklund

**Affiliations:** ^1^ Helsinki Rheumatic Diseases and Inflammation Research Group Translational Immunology Research Program University of Helsinki Helsinki University Clinicum Helsinki Finland; ^2^ Wihuri Research Institute Helsinki Finland; ^3^ Division of Orthopedics Department of Surgery Helsinki University Central Hospital Vantaa Finland; ^4^ Internal Medicine and Rehabilitation University of Helsinki and Helsinki University Hospital Helsinki Finland; ^5^ Division of Rheumatology Department of Medicine Helsinki University Hospital Helsinki Finland; ^6^ Orton Orthopaedic Hospital Helsinki Finland

**Keywords:** extracellular vesicle, high‐density lipoprotein, low‐density lipoprotein, NLRP3 inflammasome activation, oxidised lipoprotein, serum amyloid A

## Abstract

**Objectives:**

The NLRP3 inflammasome plays a key role in arterial wall inflammation. In this study, we elucidated the role of serum lipoproteins in the regulation of NLRP3 inflammasome activation by serum amyloid A (SAA) and other inflammasome activators.

**Methods:**

The effect of lipoproteins on the NLRP3 inflammasome activation was studied in primary human macrophages and THP‐1 macrophages. The effect of oxidised low‐density lipoprotein (LDL) was examined in an *in vivo* mouse model of SAA‐induced peritoneal inflammation.

**Results:**

Native and oxidised high‐density lipoproteins (HDL_3_) and LDLs inhibited the interaction of SAA with TLR4. HDL_3_ and LDL inhibited the secretion of interleukin (IL)‐1β and tumor necrosis factor by reducing their transcription. Oxidised forms of these lipoproteins reduced the secretion of mature IL‐1β also by inhibiting the activation of NLRP3 inflammasome induced by SAA, ATP, nigericin and monosodium urate crystals. Specifically, oxidised LDL was found to inhibit the inflammasome complex formation. No cellular uptake of lipoproteins was required, nor intact lipoprotein particles for the inhibitory effect, as the lipid fraction of oxidised LDL was sufficient. The inhibition of NLRP3 inflammasome activation by oxidised LDL was partially dependent on autophagy. Finally, oxidised LDL inhibited the SAA‐induced peritoneal inflammation and IL‐1β secretion *in vivo*.

**Conclusions:**

These findings reveal that both HDL_3_ and LDL inhibit the proinflammatory activity of SAA and this inhibition is further enhanced by lipoprotein oxidation. Thus, lipoproteins possess major anti‐inflammatory functions that hinder the NLRP3 inflammasome‐activating signals, particularly those exerted by SAA, which has important implications in the pathogenesis of cardiovascular diseases.

## Graphical abstract

In this study, we found that both low‐ (LDL) and high‐density lipoproteins (HDL_3_) hinder the binding of serum amyloid A (SAA) to Toll‐like receptor 4 (TLR4), which prevents SAA from activating the NLRP3 inflammasome. We further observed that native lipoproteins exert their inhibitory action on the level of inflammasome priming, but lipoprotein oxidation blocks also the NLRP3 inflammasome activation by inhibiting the complex assembly and by increasing basal autophagy. In the presence of NLRP3 inflammasome‐activating stimuli, oxidised lipoproteins possess major anti‐inflammatory functions.

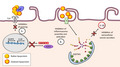

## Introduction

Serum amyloid A (SAA) is a major acute‐phase protein present in serum. It is mainly produced in the liver under the regulation of cytokines interleukin (IL)‐1, IL‐6 and tumor necrosis factor (TNF). Local expression of SAA has been demonstrated in both normal tissues^1^ as well as in inflamed tissues,^2^ such as in atherosclerotic plaques and rheumatoid synovial tissue.^3^, ^4^ Chronic inflammation is one of the key mechanisms that drive the pathogenesis of atherosclerosis.^5^ Importantly, increased plasma levels of SAA correlate with the risk of cardiovascular diseases^6^ and overexpression of SAA by viral vectors leads to acceleration of atherosclerosis in mice.^7^ SAA is a highly proinflammatory molecule, and it can induce the release of cytokines from several cell types including monocytes,^8^, ^9^ macrophages,^10^, ^11^, ^12^ neutrophils,^13^, ^14^ fibroblasts,^15^ mast cells^16^ and lymphocytes.^10^

The results of the Canakinumab Anti‐Inflammatory Thrombosis Outcome Study (CANTOS) confirmed the role of vascular inflammation and IL‐β in atherosclerosis by demonstrating that the risk of recurrent cardiovascular events can be reduced with an IL‐1β‐neutralising antibody.^17^ IL‐1β and IL‐18 are produced as precursors that need to be cleaved to become biologically active, a process mediated by inflammasomes.^18^ Numerous endogenous and exogenous danger signals capable of triggering the activation of the receptor protein nucleotide–binding domain and leucine‐rich repeat (NLR)–containing family and pyrin domain (PYD)–containing 3 (NLRP3) inflammasome have been identified.^18^ The activated NLRP3 inflammasome is a large intracellular signalling complex consisting of the receptor protein NLRP3 and the adaptor protein apoptosis‐associated speck‐like protein containing a CARD (ASC) that connects the NLRP3 to the downstream effector caspase‐1. In macrophages, two separate signals are needed for the activation of the NLRP3 inflammasome. The first signal, the priming step, triggers the synthesis of NLRP3 and pro‐IL‐1β in response to the activation of the pattern‐recognition receptors, such as Toll‐like receptors (TLRs). The second signal is needed to set off the assembly and activation of the inflammasome complex that results in proximity‐induced autocleavage of procaspase‐1.^18^ The activated caspase‐1 then processes pro‐IL‐1β and pro‐IL‐18 into their mature forms. Caspase‐1 also cleaves the effector molecule of pyroptotic cell death, gasdermin D (GSDMD). Cleavage of GSDMD releases its N‐terminal fragment that oligomerises on the cell membrane and forms pores that allow the bulk secretion of mature cytokines, whole inflammasome complexes and numerous proteins that propagate the message of the cell damage.^18^, ^19^, ^20^ In contrast, NLRP3 activators that do not activate vigorous pyroptosis first induce the vesicle‐mediated secretion of these inflammatory molecules.^18^, ^19^, ^20^, ^21^ We and others have previously shown that SAA is a strong activator of the NLRP3 inflammasome,^11^, ^12^ and interestingly, SAA has the potential to deliver both of the two signals needed for the generation of mature IL‐1β. In human macrophages, SAA activates the synthesis of pro‐IL‐1β by stimulating both TLR2 and TLR4, whereas the NLRP3 activation cascade involves signalling via the ATP‐receptor P2X7 and requires activity of the lysosome‐derived protease cathepsin B.^11^

Considering the powerful proinflammatory potential of SAA, it is obvious that regulatory mechanisms other than transcriptional regulation must exist. One such mechanism has been suggested to be the interaction of SAA with lipoproteins.^2^ The baseline level of circulating lipid‐free SAA is relatively low,^22^, ^23^ as SAA predominantly associates and circulates with the high‐density lipoprotein_3_ (HDL_3_). During the acute‐phase response, SAA replaces apoA‐I, the major apolipoprotein in the HDL_3_ particles, becoming the major HDL_3_ apolipoprotein.^24^ Multiple studies have demonstrated the ability of lipid‐free recombinant SAA as well as purified human or mouse SAA to induce proinflammatory cytokine release by cultured cells; however, the proinflammatory effects of SAA are lost when SAA is complexed with HDL or when HDL is present in the incubation medium.^2^, ^8^, ^13^, ^25^, ^26^ In the serum, most of the SAA protein is assumed to bind to HDL, but SAA can also bind with other lipoproteins or stay in a lipid‐free form.^2^, ^22^, ^23^, ^27^ For example, in patients with stable coronary artery disease, almost one fourth of the circulating SAA is associated with low‐density lipoprotein (LDL) and very low‐density lipoprotein (VLDL).^28^ Interestingly, the LDL‐SAA complex has been identified as a potent biomarker for coronary artery disease, with its sensitivity exceeding that of serum SAA or CRP alone.^28^

Here, we studied the regulation of proinflammatory activity of NLRP3 inflammasome activators by serum lipoproteins, with an emphasis on the effect of lipoproteins on the SAA‐induced activation of the NLRP3 inflammasome in human macrophages. We report that native lipoproteins, HDL_3_, and LDL, and even more surprisingly also their oxidised forms, strongly reduce the proinflammatory cytokine gene expression and secretion induced by NLRP3 inflammasome activators. We further demonstrate that oxidised LDL can inhibit the SAA‐induced IL‐1β secretion in an experimental mouse model, featuring peritoneal inflammation induced by SAA. These findings reveal a novel regulatory pathway leading to the inhibition of SAA‐induced NLRP3 inflammasome signalling and IL‐1β production *in vivo*. As inflammation and inflammasome activation in particular play a crucial role in the pathogenesis of atherosclerosis, it is vital to acknowledge both the activating and dampening pathways that contribute to the chronic inflammation present in atherosclerotic lesions.

## Results

### Native and oxidised lipoproteins inhibit the SAA‐induced cytokine secretion

**Figure 1:**
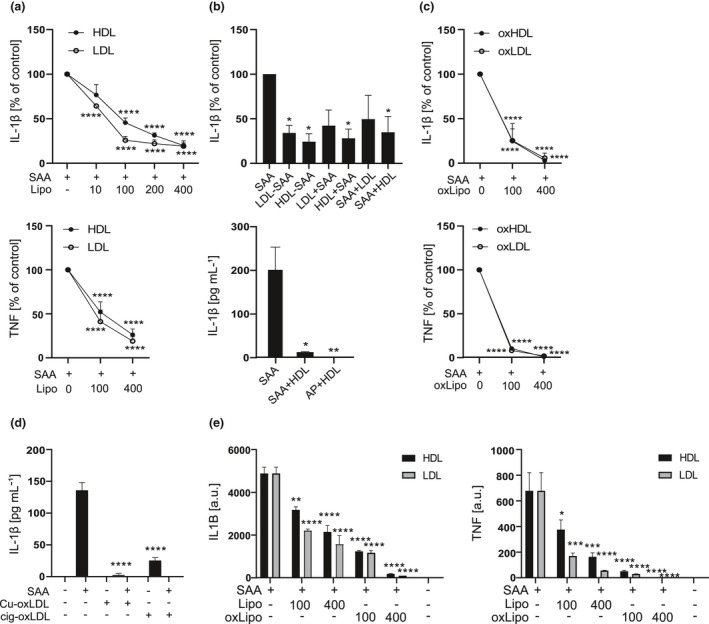
Native and oxidised lipoproteins dose dependently decrease the SAA‐induced cytokine secretion. **(a)** Primary macrophages were subjected to native HDL_3_ or LDL at indicated concentrations for 1 h and then to SAA (3 μg mL^−1^) for 18 h. Cell culture media were analysed for IL‐1β (upper panel) TNF (lower panel) concentrations by ELISA. Data are expressed relative to SAA alone (control). **(b)** Upper panel: primary macrophages were subjected to preformed LDL‐SAA or HDL_3_‐SAA complexes for 18 h, or LDL or HDL_3_ (200 µg mL^−1^) for 1 h, followed by 18‐h activation with and SAA (3 µg mL^−1^) (LDL + SAA, HDL + SAA), or activated first with SAA (3 μg mL^−1^) for 1 h, before applying LDL or HDL_3_ (200 μg mL^−1^) for 18 h (SAA + HDL, SAA + LDL). After the incubations, cell culture media were analysed for IL‐1β concentrations by ELISA. Data are expressed relative to SAA alone (control). Lower panel: Primary macrophages were incubated with plasma‐derived acute‐phase HDL (AP‐HDL), preformed HDL_3_‐SAA complex or SAA (all containing 10 μg mL^−1^ SAA), for 18 h, and cell culture media were analysed for IL‐1β concentrations by ELISA. Data are expressed as pg mL^−1^ of IL‐1β. **(c)** Primary macrophages were subjected to oxHDL_3_ or oxLDL at indicated concentrations for 1 h and then to SAA (3 μg mL^−1^) for 18 h. Cell culture media were analysed for IL‐1β (upper panel) and TNF (lower panel) concentrations by ELISA. Data are expressed relative to SAA alone (control). **(d)** Primary macrophages were subjected to copper‐oxidised and cigarette smoke‐oxidised LDL (both 200 μg mL^−1^) for 1 h and then to SAA (3 μg mL^−1^) for 18 h. Cell culture media were analysed for IL‐1β concentrations by ELISA and are expressed as pg mL^−1^. **(e)** Primary macrophages were subjected to native or oxidised HDL_3_ and LDL for 1 h and then to SAA (3 μg mL^−1^) for 5 h. *IL1B* (left panel) and *TNF* (right panel) mRNA levels were analysed by quantitative real‐time RT‐PCR and are expressed as arbitrary units (a.u.) relative to *GAPDH*. Mean values from 4 **(a)**, 3 and 2 **(b)**, 5 **(c, d)** and 2 **(e)** independent experiments are shown. *P < 0.05, **P < 0.01, ***P < 0.001, ****P < 0.0001.

First, we studied the effect of native HDL_3_, the major carrier of SAA in circulation, and LDL on the SAA‐induced release of IL‐1β and TNF in primary macrophages. Lipoproteins were added 1 h prior to the addition of SAA. As demonstrated in Figure 1a, not only HDL_3_ but also LDL significantly and dose dependently attenuated the ability of SAA to induce the release of IL‐1β and TNF. Similar results were observed when SAA was introduced to macrophages as premade complexes with HDL_3_ or LDL, or when lipoproteins were added 1 h after the addition of SAA (Figure 1b, upper panel). Also, SAA‐enriched AP‐HDL purified from plasma was unable to induce IL‐1β production (Figure 1b, lower panel).

Oxidised lipoproteins play a significant role in the pathogenesis of atherosclerosis.^5^ Therefore, we next studied whether oxidation of lipoproteins modifies their ability to inhibit the SAA‐induced cytokine production. Both LDL and HDL_3_ were oxidised by Cu^2+^, and the level of lipid peroxidation was determined by TBARS analysis. The levels were typically 20‐30 nmol malonaldehyde (MDA) mg^−1^ protein for LDL and 10–20 nmol MDA mg^−1^ protein for HDL_3_. As demonstrated in Figure 1c, inhibition of the SAA‐induced IL‐1β and TNF secretion by oxidised lipoproteins was even more pronounced than that exhibited by their native counterparts. Furthermore, LDL oxidised by cigarette smoke‐treated PBS also inhibited the SAA‐induced IL‐1β release (Figure 1d), verifying that the inhibition is not because of properties specific to copper‐oxidised LDL. As SAA is capable of providing both the priming and activating signal for the NLRP3 inflammasome,^11^, ^12^ we continued by studying whether lipoproteins inhibit the SAA‐induced expression of proinflammatory proteins in primary macrophages. Figure 1e shows that addition of both native and oxidised HDL_3_ and LDL prior to SAA activation dose dependently reduced mRNA levels of both inflammasome target protein *IL1B* and the prototypical proinflammatory protein *TNF*.

### Uptake of lipoproteins is not required for the inhibition of the activity of SAA

**Figure 2:**
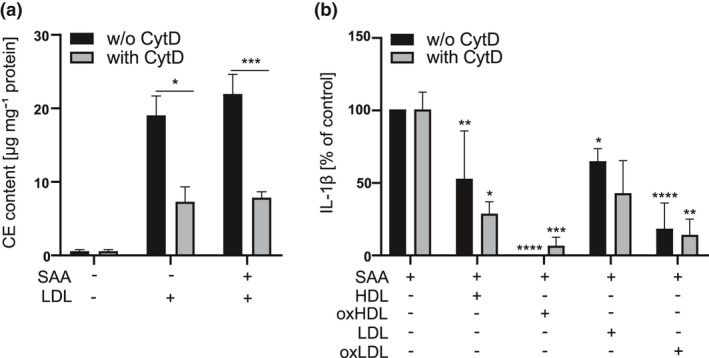
Uptake of lipoproteins is not a prerequisite for the inhibition of SAA‐induced IL‐1β secretion. **(a)** Primary macrophages were subjected to LDL (1 mg mL^−1^) or preformed LDL‐SAA complexes (1 mg mL^−1^ LDL, 25 μg mL^−1^ SAA) with or without cytochalasin D (1 μg mL^−1^) for 18 h. After this, cellular lipids were extracted and analysed by thin layer chromatography. The protein concentration was determined by the Lowry method. The data are expressed as μg cholesterol ester (CE) per mg cellular protein. **(b)** Primary macrophages were subjected to preincubation with native or oxidised HDL_3_ and LDL (200 μg mL^−1^) and cytochalasin D (1 μg mL^−1^) for 1 h and then exposed to SAA (3 μg mL^−1^) for 18 h. After the incubation, cell culture media were analysed for IL‐1β concentrations by ELISA. Data are expressed relative to respective control (SAA activation). Mean values shown are from 2 **(a)** or 3 **(b)** independent experiments. *P < 0.05, **P < 0.01, ***P < 0.001, ****P < 0.0001.

Uptake of oxidised lipoproteins could initiate intracellular signalling resulting in a diminished proinflammatory response. Therefore, we next studied whether internalisation of lipoproteins is required for their inhibitory effect. This was assessed by using cytochalasin D, as the phagocytosis of lipoproteins is dependent on actin polymerisation.^29^, ^30^ As shown in Figure 2a, cytochalasin D inhibited the uptake of LDL as well as the uptake of LDL‐SAA complexes in primary macrophages as reflected by the decrease in the intracellular content of cholesterol esters (CE) in the presence of cytochalasin D. However, inhibition of lipoprotein phagocytosis by cytochalasin D had no effect on the ability of native or oxidised lipoproteins to inhibit the SAA‐induced secretion of IL‐1β (Figure 2b), suggesting that internalisation of lipoproteins is not required for their inhibitory effect.

### Inhibition of IL‐1β secretion requires co‐presence of native but not oxidised lipoproteins with SAA

**Figure 3:**
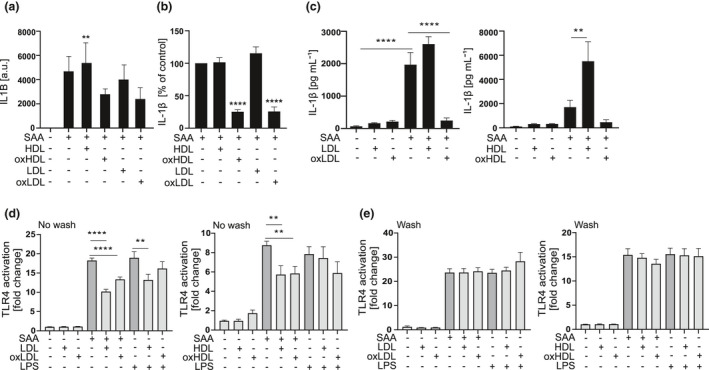
Inhibition by oxidised lipoproteins does not require binding to SAA. **(a)** Primary macrophages were subjected to native or oxidised HDL_3_ and LDL (400 μg mL^−1^) for 1 h, washed and changed into fresh SFMM containing SAA (3 μg mL^−1^), and incubation was continued for 5 h. *IL1B* mRNA levels were analysed by quantitative real‐time RT‐PCR relative to *GAPDH*. Data are expressed as arbitrary units (a.u.) relative to *GAPDH*. **(b)** Primary macrophages were subjected to native or oxidised HDL_3_ and LDL (400 μg mL^−1^) for 1 h, washed and changed into fresh SFMM containing SAA (3 μg mL^−1^) and incubated for 18 h. Cell culture media were analysed for IL‐1β concentrations by ELISA. **(c)** THP‐1 macrophages were subjected to native or oxidised HDL_3_ and LDL (200 μg mL^−1^) for 1 h and then to SAA (3 μg mL^−1^) for 5 h. Secretion of IL‐1β was analysed from cell culture media by ELISA. HEK‐blue TLR4 reporter cells were changed to SFMM before addition of native or oxidised HDL_3_ and LDL (lipoproteins), **(d)** no wash group, or after 1‐h lipoprotein activation, before SAA was applied, **(e)** wash group, to remove the lipoproteins. SEAP activity was detected from supernatants 24 h after addition of lipoproteins. The data are presented as fold changes compared to separate untreated cells. Data are means from 2 **(a)** or at least 3 **(b)** or 4 **(c–e)** independent experiments. **P < 0.01, ****P < 0.0001.

To elucidate the mechanism by which lipoproteins inhibit the SAA‐mediated IL‐1β secretion, we next tested whether co‐presence of SAA and lipoproteins is required for the inhibitory effect. Primary macrophages were first incubated in the presence of native and oxidised lipoproteins for 1 h, after which they were thoroughly washed to remove lipoproteins, and then, inflammasome activation was induced by a 5‐h incubation with SAA. As shown in Figure 3a and b, the ability of native LDL and HDL_3_ to inhibit the *IL1B* expression (Figure 3a) and secretion of IL‐1β (Figure 3b) was lost. Similarly, removal of oxidised lipoproteins also reversed their ability to significantly inhibit the expression of *IL1B* (Figure 3a). However, the SAA‐induced secretion of IL‐1β continued to be significantly reduced in macrophages preincubated with oxidised lipoproteins even when oxidised lipoproteins were removed before activation with SAA (Figure 3b).

The results above indicated that lipoproteins have to be co‐present with SAA in order to inhibit the SAA‐induced expression of *IL1B* (Figure 1e). This prompted us to study whether the inhibitory effects can be attributed solely to the reduced NLRP3 inflammasome priming or whether lipoproteins also interfere with the inflammasome activation step. To address this, we activated PMA‐treated THP‐1 macrophages with SAA in the presence of lipoproteins. PMA treatment induces the activation of nuclear factor κB (NF‐κB) in THP‐1 cells, and therefore, differentiated THP‐1 macrophages express inflammasome components *IL1B* and *NLRP3* in a stable manner.^31^ In line with this, additional priming of THP‐1 macrophages with LPS did not increase the SAA‐induced secretion of IL‐1β (Supplementary figure 1a). As demonstrated in Figure 3c, in stably primed THP‐1 macrophages, native lipoproteins did not inhibit SAA‐induced inflammasome activation; instead, native HDL had an additive effect on the SAA‐induced IL‐1β release. In contrast, oxidised lipoproteins, oxidised LDL (oxLDL) in particular, inhibited the SAA‐induced IL‐1β secretion also in these cells. However, oxidised lipoproteins did not inhibit the pyroptotic cell death, as reflected by secretion of LDH (Supplementary figure 1b).

Previous observations suggesting that the inflammatory properties of SAA are lost when it is bound to HDL^2^, ^8^, ^13^, ^25^, ^26^ and the present results indicating that co‐presence with SAA is required for the inhibitory effect of native lipoproteins imply that binding of native lipoproteins to SAA may inhibit its interactions with TLR4 and TLR2.^11^ To test this, we studied the impact of lipoproteins on binding of SAA to TLR4 in HEK‐TLR4 cells. Lipoproteins were added to the cells for one hour and thereafter either left there or removed before addition of SAA or LPS. Both native and oxidised lipoproteins significantly inhibited the SAA‐induced TLR4 activation, with the effect of native LDL being more pronounced than that of oxLDL (Figure 3d). Removal of lipoproteins before activation of the cells with SAA or LPS completely abolished the inhibitory effects (Figure 3e). In conclusion, these results suggest that lipoproteins reduce the interaction of SAA with TLR4, and thereby inhibit both the priming and activation of the NLRP3 inflammasome. Furthermore, the inhibitory effect of native lipoproteins appears to result mostly from the reduced NLRP3 inflammasome priming; that is, they reduce the expression of pro‐IL‐1β, while the inhibitory effects of oxidised lipoproteins extend beyond priming.

### Lipoproteins inhibit the NLRP3 inflammasome activation by different activators and activation of different types of inflammasomes in THP‐1 macrophages

**Figure 4:**
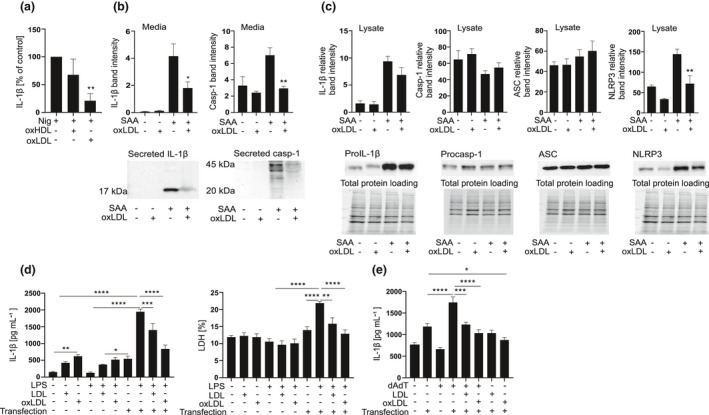
Oxidised lipoproteins inhibit activation of NLRP3, noncanonical and AIM2 inflammasomes. **(a)** THP‐1 macrophages were subjected to oxidised HDL_3_ or LDL (200 μg mL^−1^) for 1 h and then to nigericin (4 μm) for 1 h. Cell culture media were analysed for IL‐1β concentrations by ELISA. Data are expressed relative to nigericin alone (control). **(b)** THP‐1 macrophages were subjected to oxLDL (200 μg mL^−1^) for 1 h and then to SAA (3 µg mL^−1^) for 5 h. The secreted mature IL‐1β (17 kDa) and caspase‐1 (20 kDa) were determined by Western blot from cell culture media. **(c)** The intracellular levels of pro‐IL‐1β (37 kDa), procaspase‐1 (45 kDa), ASC (22 kDa) and NLRP3 (108 kDa) were assessed from Western blots of the cell lysates. Data are expressed as band intensities relative to total protein loading per lane. **(d)** THP‐1 macrophages were subjected to LDL or oxLDL (200 μg mL^−1^) for 1 h and then transfected with ultrapure LPS (2 µg mL^−1^) for 6 h. Cell culture media were analysed for IL‐1β concentrations by ELISA (left panel) and for concentrations of LDH (right panel) by cell death assay. **(e)** THP‐1 macrophages were subjected to LDL or oxLDL (200 μg mL^−1^) for 1 h and then transfected with poly(dA:dT) for 5 h. Cell culture media were analysed for IL‐1β concentrations by ELISA. The mean values shown are from 3 **(a)** and 4 **(b–e)** experiments, Western blots and the corresponding loading shown are representatives of 4 individual experiments. *P < 0.05, **P < 0.01, ***P < 0.001, ****P < 0.0001.

Next, we studied whether the inhibitory effect of oxidised lipoproteins is limited to SAA by studying their effect on the nigericin‐induced NLRP3 inflammasome activation in THP‐1 macrophages. OxLDL inhibited the nigericin‐induced secretion of IL‐1β (Figure 4a), but the effect of oxidised HDL (oxHDL) was not significant. We confirmed the inhibitory effect of oxLDL on the SAA‐induced NLRP3 inflammasome activation by measuring the levels mature IL‐1β and active caspase‐1 in the cell culture supernatants by Western blot (Figure 4b). OxLDL had no significant effect on protein expression of pro‐IL‐1β, procaspase‐1 or ASC in THP‐1 macrophages; however, protein expression of NLRP3 was downregulated by the oxLDL treatment (Figure 4c). To study whether the inhibitory effects of LDLs extend beyond the NLRP3 inflammasome, we studied their effect on the activation of noncanonical and AIM2 inflammasomes. For noncanonical inflammasome activation, we transfected LPS into THP‐1 macrophages. Both LDL types slightly increased the secretion of IL‐1β, but when combined with the activation of noncanonical inflammasome, oxLDL and to lesser extent also native LDL, inhibited the secretion of IL‐1β (Figure 4d, left panel). Both lipoproteins also inhibited the activation of the noncanonical inflammasome that induces pyroptotic cell death, as reflected by the lower release of LDH (Figure 4d, right panel). Finally, we studied the effect of native and oxidised LDL on the AIM2 inflammasome activation, which was induced by transfection of synthetic double‐stranded DNA, poly(dA:dT), in THP‐1 macrophages. Again, both LDL types inhibited the secretion of IL‐1β induced by AIM2 inflammasome activation (Figure 4e). In conclusion, these results imply that oxLDL is a general inhibitor of inflammasome activation.

### Oxidised LDL inhibits NLRP3 inflammasome activation induced also by activators other than SAA in primary macrophages

**Figure 5:**
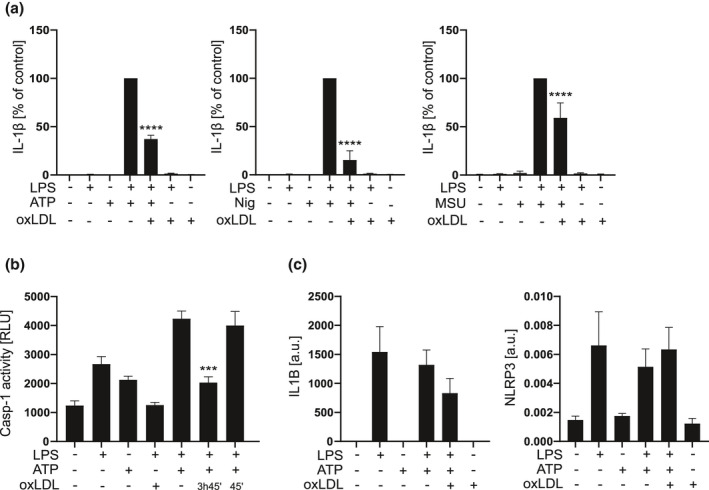
Oxidised LDL inhibits a wide variety of NLRP3 inflammasome activators. **(a)** Primary macrophages were primed with LPS for 3 h, then oxidised LDL 400 µg mL^−1^ was added for 3 h, after which the cells were activated with ATP 5 mm for 30 min, nigericin 10 μm for 1 h or MSU crystals 200 µg mL^−1^ for 4 h. Secretion of IL‐1β was analysed from cell culture media by ELISA. Data are expressed relative to activation alone (control). **(b)** Primary monocytes were primed with LPS for 4 h 45 min, during which oxLDL 400 µg mL^−1^ was added for indicated times and the cells were activated with ATP for the last 45 min of the incubation. Caspase‐1 activity was analysed from culture media and is expressed as relative luminescence units (RLU). **(c)** Primary macrophages were primed with LPS for 3 h, then subjected to oxLDL for 3 h and activated with ATP (5 mm, 30 min). The expression of *IL1B* and *NLRP3* mRNA was analysed by quantitative real‐time RT‐PCR and is expressed as arbitrary units (a.u.) relative to *GAPDH*. The mean values shown are from 7 **(a)** and 4 **(b, c)** experiments. ***P < 0.001, ****P < 0.0001.

To verify the inhibitory effects of oxLDL on human primary cells, we activated LPS‐primed primary macrophages with other, well‐recognised NLRP3 inflammasome activators. As expected, addition of oxLDL significantly inhibited the secretion of IL‐1β induced by ATP, nigericin and MSU (Figure 5a). The inhibitory effect of oxLDL was not restricted to macrophages, but oxLDL also inhibited the NLRP3 inflammasome‐dependent activation of caspase‐1 in primary monocytes (Figure 5b). However, addition of oxLDL had to take place before the inflammasome activation step in monocytes, as oxLDL treatment given simultaneously with activation had no inhibitory effect (Figure 5b).

ATP, nigericin and MSU can only induce the assembly and activation of NLRP3 inflammasome but cannot deliver the priming signal, and therefore, LPS was used to prime the cells before activation. Since it has been previously shown that oxLDL treatment preceding LPS activation selectively inhibits transcription of a subset of proinflammatory genes in mouse macrophages,^32^ we studied the effect of oxLDL treatment applied 3 h after LPS on primary macrophages. We analysed the expression of inflammasome components *NLRP3* and *IL1B* (Figure 5c), and a selected set of other genes, including *NFKBIA, IRF1, IL‐6, CXCL10* and *CCL2,* all of which depend on the activation of transcription factor NF‐κB (Supplementary figure 2). In addition to NF‐κB, other signalling cascades also affect the expression of these genes; for example, *IL1B*, *NFKBIA*, *IL‐6* and *CCL2* are canonical IL‐1 target genes, and therefore, their expression also depends on the IL‐1 receptor activation by extracellular IL‐1β.^33^ OxLDL did not inhibit the LPS‐induced transcription of *IL1B, NLRP3* or *NFKBIA* (Figure 5c and Supplementary figure 2), implicating that when the priming signal is introduced before oxLDL, gene expression induced via NF‐κB is not considerably downregulated. The expression of *CXCL10, IRF1, IL‐6* and *CCL2* was downregulated, but the change was not significant (Supplementary figure 2). Therefore, it is plausible that also the inhibition of IL‐1β secretion by the oxLDL treatment contributed to the reduced gene expression. We also studied whether oxLDL activates PPAR‐γ or PPAR‐α, both of which reduce the TLR4/NF‐κB pathway activation.^32^, ^34^ However, as anticipated based on the modest effect of oxLDL on the expression of NF‐κB‐dependent genes, neither PPARγ (T0070907) nor PPARα (GW6471) antagonists were able to reverse the inhibitory effect of oxLDL in PMA‐primed THP‐1 macrophages (Supplementary figure 3a). In conclusion, oxLDL generally inhibits the second activating step of NLRP3 inflammasome activation in human primary macrophages.

### Oxidised LDL impairs NLRP3 inflammasome assembly and the inflammasome‐induced extracellular vesicle secretion

**Figure 6:**
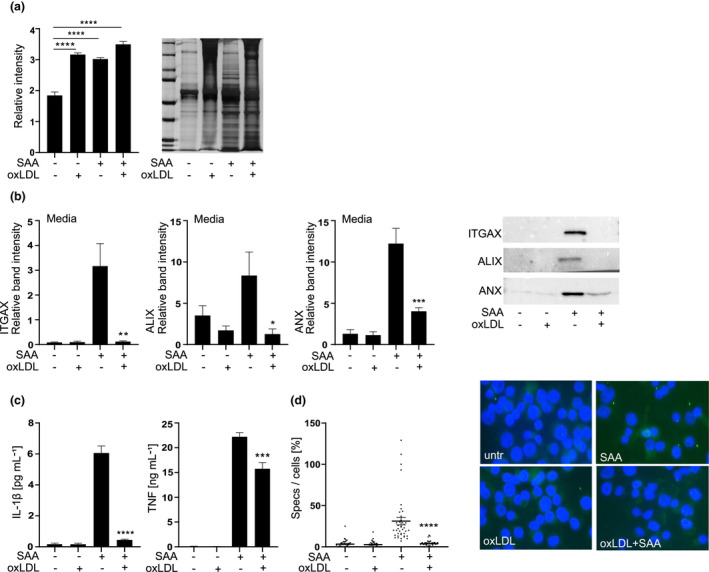
Oxidised LDL inhibits the NLRP3 inflammasome‐induced vesicle secretion and ASC oligomerisation. **(a)** THP‐1 macrophages were treated with oxLDL 200 µg mL^−1^ for 1 h and challenged with SAA (3 μg mL^−1^) for 5 h. Silver‐stained SDS‐PAGE gel was used to estimate the total protein secretion. On the left average of the relative lane volumes are shown, on the right representative silver staining is shown. **(b)** Secretion of ITGAX, alix and annexin‐1 (ANX) was analysed by Western blotting. Equal volumes of media were loaded, on the right representative blots are shown. **(c)** Secretion of IL‐1β and TNF was analysed from media samples used for analysis of extracellular vesicle secretion and are expressed as ng mL^−1^ of IL‐1β (left panel) or TNF (right panel). **(d)** THP‐1 macrophages were pretreated with oxLDL 400 µg mL^−1^ for 1 h and activated with SAA (3 μg mL^−1^) for 5 h and ASC oligomerisation was visualised by stainings, ASC (green) and nuclei (blue, DAPI). On the left: speck formation was quantified by counting the number of specks per field, which was divided by the number of nuclei per field. On the right: stainings are representatives of four independent experiments. Mean values shown are from 4 **(a–d)** independent experiments. *P < 0.05, **P < 0.01, ***P < 0.001, ****P < 0.0001.

It is not fully understood how the leaderless mature IL‐1β is secreted, but vesicle‐mediated secretion is thought to initiate the secretion in nonpyroptotic cells, which is later followed by the bulk secretion of IL‐1β through GSDMD pores during pyroptosis.^20^ Under our experimental conditions, pyroptosis was not significantly induced in THP‐1 macrophages, corresponding to the secretion of IL‐1β from nonpyroptotic cells (Supplementary figure 1b). In primary macrophages, inflammasome activation has been shown to be associated with enhanced vesicle secretion,^21^ and therefore, we studied the impact of oxLDL treatment on extracellular vesicle secretion. OxLDL did not reduce the overall protein secretion in THP‐1 macrophages compared with SAA alone, as shown by silver stainings (Figure 6a). However, Western blots of extracellular vesicle proteins showed a specific inhibition of extracellular vesicle secretion (Figure 6b). As shown in the analysis of the cell culture media from the same cells, the secretion of IL‐1β was reduced concomitantly with vesicle secretion (Figure 6c, left panel). Secretion of TNF was also reduced but to a much lesser extent (Figure 6c, right panel), which may be merely because of reduction in its transcription given that secretion of TNF is independent of inflammasome activation and vesicle secretion. Next, we assessed the impact of oxLDL on the NLRP3 inflammasome assembly. As reflected by the reduced ASC oligomerisation, i.e., reduced ASC speck formation (Figure 6d), oxLDL prevented the inflammasome complex formation. In conclusion, our results imply that oxLDL does not inhibit protein secretion per se but specifically inhibits the activation of the NLRP3 inflammasome and the secretion of extracellular vesicles associated with the inflammasome activation.

### Oxidised lipoproteins induce autophagy, which limits NLRP3 inflammasome activation

**Figure 7:**
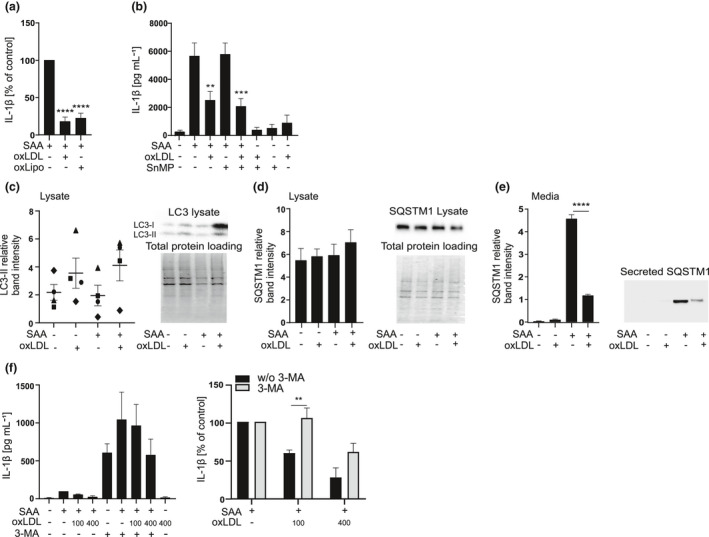
Autophagy contributes to the inhibitory effect of oxidised LDL. **(a)** Primary macrophages were subjected to oxLDL or microemulsion particles prepared of oxLDL (oxLipo) (both containing 450 μg mL^−1^ cholesterol) for 1 h and then to SAA (3 μg mL^−1^) for 18 h. Cell culture media were analysed for IL‐1β concentrations by ELISA. Data are expressed relative to SAA alone (control). **(b)** THP‐1 macrophages were pretreated with SnMP 5 mm for 3 h, and then, oxLDL 200 µg mL^−1^ was added for 1 h, finally cells were challenged with SAA 3 mg mL^−1^ for 5 h. Cell culture media were analysed for IL‐1β concentrations by ELISA. **(c)** THP‐1 macrophages were subjected to oxLDL (200 μg mL^−1^) for 1 h and then to SAA for 5 h. The intracellular levels of LC3‐II (17 kDa) and **(d)** SQSTM1 (62 kDa) were assessed from Western blots of the cell lysates, and **(e)** secretion of SQSTM1 was analysed form concentrated media by Western blotting. Data showing the independent experiments as individual symbols or as means of experiments are expressed as band intensities relative to total protein loading per lane. **(f)** THP‐1 macrophages were pretreated with 3‐MA 5 mm and oxLDL 100 µg mL^−1^ or 400 µg mL^−1^ for 1 h and activated with SAA 3 mg mL^−1^ for 5 h. On the left: secretion of IL‐1β was analysed by ELISA. On the right: the effect of 3‐MA is presented as per cent change compared to respective activated control. Mean values shown are from 3 **(a)** and 4 **(b–f)** independent experiments. **P < 0.01, ***P < 0.001, ****P < 0.0001.

To further study the mechanism by which oxidised lipoproteins inhibit the NLRP3 inflammasome activation, we next proceeded to determine whether the protein or the lipid component of the lipoprotein particles is responsible for the inhibition. For this purpose, microemulsion particles were prepared from lipids extracted from oxLDL. Primary macrophages were subjected to these particles or to intact oxLDL, containing equal amounts of cholesterol as microemulsion particles, and then challenged with SAA. The microemulsion particles inhibited the release of IL‐1β as efficiently as did the intact oxLDL (Figure 7a). Oxidised lipids can induce the expression of antioxidant defence stress proteins in macrophages, among which is heme oxygenase‐1 (HO‐1), which we have previously shown to inhibit the inflammasome activation.^35^ Although oxLDL induced the expression of *HMOX1* in primary macrophages (Supplementary figure 3b), treatment of THP‐1 macrophages with an HO‐1 enzymatic inhibitor, tin mesoporphyrin (SnMP), did not reverse the inhibitory effect of oxLDL (Figure 7b). Furthermore, the inhibitory effect of oxLDL was not dependent on the formation of reactive oxygen species (ROS) as addition of mitoTEMPO, a specific inhibitor of mitochondrial ROS, or *N*‐acetyl l‐cysteine (NAC), an inhibitor of NADPH ROS, did not have an impact on the inhibitory effect of oxLDL (Supplementary figure 3c and d).

Previously, OxLDL has been shown to induce autophagy in mouse BMDMs,^36^ and since autophagic flux inhibits the activation of the NLRP3 inflammasome,^37^, ^38^ we studied the effect of oxLDL on autophagosome formation, which is characterised by the conversion of microtubule‐associated protein light chain 3‐I (LC3‐I) to LC3‐II.^20^ In SAA‐activated THP‐1 macrophages, oxLDL consistently increased the levels of LC3‐II, but because of the variability in these levels, this difference was not statistically significant (Figure 7c). We also analysed the intracellular levels of SQSTM1, which, according to current paradigm, decrease upon activation of autophagy. However, oxLDL did not impact the SQSTM1 levels (Figure 7d). The role of autophagy in the activation of NLRP3 inflammasome is somewhat ambiguous. Basal autophagy is considered to inhibit the NLRP3 inflammasome activation by degrading inflammasome components and ROS‐producing mitochondria; however, induced autophagy following inflammasome activation is required for the secretion of mature IL‐1β.^20^ Therefore, we next studied the levels of SQSTM1 in cellular supernatants. Interestingly, we found a robust secretion of SQSTM1 upon SAA‐induced NLRP3 inflammasome activation, and this secretion was significantly blocked by the oxLDL (Figure 7e). Based on this as well as previously published studies, we propose that NLRP3 inflammasome activation induces the initial secretion of IL‐1β in extracellular vesicles, which is marked by a robust secretion of SQSTM1 that reduces intracellular levels of SQSTM1. To the best of our knowledge, secretion of SQSTM1 during the NLRP3 inflammasome activation has not been previously reported. We further studied whether inhibition of autophagic sequestration by phosphoinositide 3‐kinase inhibitor (PI3K) 3‐methyl adenine (3‐MA) would reverse the inhibitory effect of oxLDL (Figure 7f, left panel). Interestingly, 3‐MA fully reversed the inhibitory effect of 100 µg mL^−1^ oxLDL and partially reversed the effect of higher 400 µg mL^−1^ oxLDL (Figure 7f, right panel). In conclusion, only the lipid fraction of oxLDL particles is required for the inhibition of the SAA‐induced IL‐1β production, and this inhibitory effect is at least partially mediated by increased basal autophagy.

### OxLDL inhibits the SAA‐induced IL‐1β secretion *in vivo*

**Figure 8:**
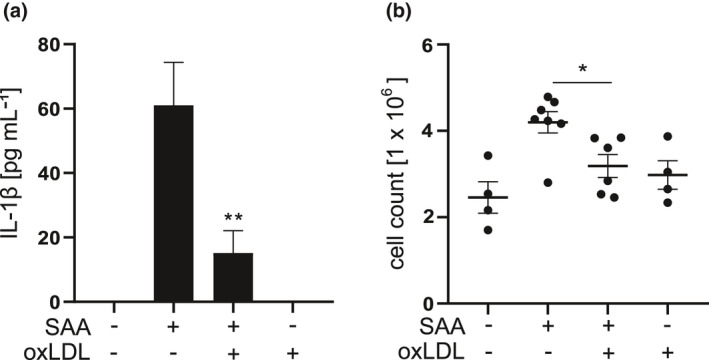
OxLDL inhibits SAA‐induced peritoneal inflammation and secretion of IL‐1β in mice. C57BL/6J mice were given an i.p. injection of oxLDL (400 μg 300 μL^−1^ per mouse) or PBS (300 μL per mouse) and 1 h later an injection of SAA (30 μg 300 μL^−1^ per mouse) or vehicle (PBS, 300 μL per mouse). After 4 h, the mice were terminally anesthetised, peritoneal cavities were lavaged and the peritoneal fluid was analysed for **(a)** IL‐1β concentration by ELISA and **(b)** number of peritoneal cells. Data are means from the number of mice as follows: 4 (PBS only), 7 (SAA only), 6 (oxLDL + SAA) and 4 (oxLDL only). *P < 0.05, **P < 0.01.

Finally, we studied whether oxidised lipoproteins also inhibit the SAA‐induced IL‐1β production *in vivo*. To study this, SAA was injected with or without oxLDL into the peritoneal cavities of C57BL/6J mice. Intraperitoneal injection of SAA increased the level of IL‐1β (Figure 8a) as well as the number of leukocytes in the peritoneal fluid (Figure 8b), as determined 4 h after injection. Importantly, injection of oxLDL into the peritoneal cavity prior to SAA clearly diminished the SAA‐induced peritoneal inflammation. These findings revealed that the inhibitory effect of oxidised lipoproteins observed in cell culture experiments can occur also *in vivo*.

## Discussion

SAA is a powerful proinflammatory mediator, capable of activating the NLRP3 inflammasome with ensuing production of the key proinflammatory cytokine IL‐1β.^11^ Considering that the serum levels of SAA can increase up to 1000‐fold during inflammation, it is evident that stringent SAA‐regulating mechanisms must exist in serum and tissues. Here, we demonstrate that native and oxidised lipoproteins, HDL_3_, and LDL, play a key role in the regulation of the SAA‐induced secretion of proinflammatory cytokines, IL‐1β in particular. Furthermore, oxidised LDL inhibited inflammasome activation induced by SAA as well as by all other inflammasome activators tested. Native and oxidised lipoproteins inhibited IL‐1β secretion by distinct mechanisms; native lipoproteins inhibited only the priming of the NLRP3 inflammasome, whereas oxidised LDL also inhibited the NLRP3 inflammasome activation.

In our study, both native HDL_3_ and LDL dose dependently inhibited the SAA‐induced production of IL‐1β and TNF in primary macrophages (Figure 1a). In agreement with this, Shridas *et al*. have shown that native HDL inhibits the SAA‐induced transcription of *Il1b* and activation of the NLRP3 inflammasome in mouse macrophages.^39^ HDL has also been shown to inhibit inflammasome activation induced by cholesterol crystals that are relevant in the context of cardiovascular diseases,^40^, ^41^ as well as other particles and soluble activators including ATP, nigericin and synthetic AIM2 inflammasome activator poly(dA:dT)^40^ in human macrophages and whole blood. Furthermore, HDL has been reported to inhibit the SAA‐induced production of IL‐1β, TNF and monocyte tissue factor in PBMCs.^10^, ^26^, ^42^ In the present study, native HDL_3_ and LDL inhibited the SAA‐induced expression of *IL1B* and *TNF* (Figure 1e) but had no effect on the activation of NLRP3 inflammasome (Figure 3c). Their inhibitory effect was dependent on the co‐presence with SAA, while their uptake was not required (Figures 2 and 3). Complex formation between SAA and lipoproteins could prevent SAA from interacting with its cell surface receptors or, alternatively, internalisation of SAA‐lipoprotein complex with ensuing degradation of SAA could result in the reduced activity of SAA. Indeed, complexation of SAA and lipoproteins or commercial AP‐HDL prevented the SAA‐induced secretion of IL‐1β (Figure 1b). It is likely that lipoproteins bind to SAA as their incubation in the presence of SAA significantly inhibited SAA‐induced TLR4 activation (Figure 3d). Together, these findings suggest that the inhibitory effect of native LDL and HDL_3_ is based on their extracellular interaction with SAA, which results in diminished interactions of SAA with its cell surface receptors.

OxLDL possesses many proinflammatory properties, and lipoprotein oxidation is considered to play a major role in the development of atherosclerosis.^2^ Considering this, it was surprising that oxLDL and also oxHDL_3_ strongly suppressed the SAA‐induced secretion of IL‐1β (Figures 1c and 3c). Unlike the inhibition induced by native lipoproteins, the inhibition by oxidised lipoproteins was not dependent on the complex formation between SAA and the lipoproteins, as the inhibition persisted in cells from which the lipoproteins had been removed before SAA‐induced activation (Figure 3b). Both lipoprotein types inhibited the SAA‐induced *IL1B* expression, but only the oxidised lipoproteins inhibited also the activation of the NLRP3 inflammasome, as shown in the experiments with THP‐1 macrophages (Figures 3c and 4a). These data suggest that the inhibition of the secretion of mature IL‐1β cannot be merely because of the inhibition of inflammasome component expression, since THP‐1 macrophages are stably primed by the PMA treatment. These results were further confirmed by the results, showing that oxLDL can also inhibit the NLRP3 inflammasome activation induced by other well‐recognised inflammasome activators, including ATP, nigericin and MSU crystals (Figure 5a). Based on our findings, the inhibitory effect of oxLDL appears to target a process upstream of NLRP3 inflammasome oligomerisation and is common for all used NLRP3 activators. Further, oxidised and, to a lesser extent, also native LDLs inhibited the secretion of IL‐1β induced by the activation of the noncanonical and AIM2 inflammasomes (Figure  4d, e). While it is possible that LDLs affect the potassium efflux, which precedes the NLRP3 inflammasome activation by most of the NLRP3 activators, it is not needed for the activation of noncanonical or AIM2 inflammasomes. As both LDL types inhibited all studied inflammasomes, it is likely that mechanisms other than the inhibition of potassium efflux mediate the inhibitory effect.

OxLDL was shown to block the secretion of extracellular vesicles, which is thought to induce the initial unconventional secretion of IL‐1β, while the overall protein secretion was not reduced with oxLDL treatment (Figure  6a, b). In addition to vesicle‐mediated secretion, IL‐1β is also considered to be secreted via pyroptosis, through GSDMD pores.^20^ In a recent study, caspase‐1 was shown to cleave an endosome docking and fusion machinery protein, the early endosomal autoantigen 1, which is required for the secretion of mature IL‐1β but not that of LDH or inflammasome components excluding caspase‐1.^43^ These results are in accordance with our results showing that oxLDL does not inhibit pyroptosis or the total protein secretion, while it efficiently blocks IL‐1β secretion. Based on our present results, lipoproteins appear to affect processes that precede the cleavage of pro‐IL‐1β. Native lipoproteins act mainly on the level of priming and oxidised lipoproteins on the level of NLRP3 inflammasome assembly and caspase‐1 activation, without affecting the NLRP3‐induced pyroptosis. To further elucidate the intracellular signalling events leading to the inhibition of the NLRP3 inflammasome activation, we also investigated the involvement of oxidative stress and PPARs, but their inhibition did not reverse the effect of oxidised lipoproteins.

Autophagy has been implicated in the pathogenesis of atherosclerosis and has been shown to inhibit the NLRP3 inflammasome activation and apoptosis and to relieve oxidative stress.^44^ Macrophage‐specific deletion of autophagy proteins has been shown to lead to an increased formation of atherosclerotic lesions and secretion of IL‐1β *in vivo* in an ApoE^‐/‐^ mouse model of atherosclerosis.^45^ As lipid accumulation in macrophages induces autophagy, we explored whether oxLDL could induce autophagy and whether autophagy could be involved in the inhibitory effect. Autophagosome formation was increased in the presence of oxLDL, although this finding was not statistically significant (Figure 7c). We could not detect any significant changes in the intracellular levels of SQSTM1. However, we did detect a robust secretion of SQSTM1 upon inflammasome activation (Figure 7e). This secretion was significantly reduced in the presence of oxLDL, suggesting that oxLDL can reduce the secretory autophagy. Furthermore, inhibition of autophagy reversed the anti‐inflammatory effects of 100 µg mL^−1^ oxLDL (the lower concentration studied). Therefore, inhibition of autophagy seems to play a significant role in the inhibition of NLRP3 inflammasome activation by oxidised lipoproteins, but it is likely that other mechanisms are also involved. Recently, Thi Tran *et al*. showed that several NLRP3 inflammasome activators lower the intracellular pH and the level of ATP, and that while a decline in intracellular ATP or pH within an optimal range induces maximal IL‐1β release, their excessive decline suppresses IL‐1β release.^46^ Therefore, the effect of two inflammasome activators that simultaneously boost the same pathway may be inhibitory, which might explain the inhibitory effect of oxLDL in the presence of an inflammasome activator.

It has been previously reported that minimally modified LDL and/or oxLDL or oxidised lipoprotein particles isolated from human atherosclerotic lesions induce, in addition to the expression of *IL1B*,^47^ also the release of IL‐1β via NLRP3 inflammasome activation.^48^, ^49^, ^50^, ^51^ Uptake of oxLDL, long incubation of the cells in the presence of oxLDL and, in some cases, foam cell formation have been shown to be prerequisites for the inflammasome activation.^49^, ^50^ It should be pointed out that, in some experiments, we also detected a small secretion of IL‐1β in response to lipoproteins alone. Sheedy *et al*. have shown that uptake of oxLDL via CD36 directs it into lysosomes where it forms cholesterol crystals,^50^ potent activators of the NLRP3 inflammasome.^48^, ^52^ In contrast, however, the inhibitory effect of oxLDL observed in our study was independent of lipoprotein uptake (Figure 2). Furthermore, the inhibitory effect was fully induced in primary macrophages after a short (1–3 h) preincubation before the induction of inflammasome activation with ATP, nigericin, MSU crystals or SAA, that is times that are presumably too short for any extensive foam cell formation or cholesterol crystallisation to take place. It should be stressed that the results of the present study do not contradict with the previous results showing a relatively weak activation of inflammasome by oxidised lipoproteins. Our results may merely indicate that when macrophages are challenged with a strong inflammasome activator, such as SAA, the presence of oxidised lipoproteins exert an inhibitory effect instead of activation.

Unsaturated fatty acids, oleate and linoleate, have been reported to inhibit the NLRP3 inflammasome *in vitro*.^53^ In aqueous environments, such as blood, the hydrophobic free fatty acids are bound to plasma proteins. In this study, we showed that the lipid fraction of oxLDL inhibited the NLRP3 inflammasome activation similar to intact lipoproteins (Figure 7a). Native LDL and HDL_3_ particles contain small amounts of free fatty acids, while a particularly extensive oxidation, such as used in this study, can lead to degradation of lipoprotein phospholipids and generation of lysophospholipids and free fatty acids.^54^, ^55^ In both LDL and HDL, linoleate and oleate are the major fatty acid components of the surface phospholipids. Thus, it is possible that the inhibitory effects of oxLDL are mediated by their free fatty acids. Free fatty acids can also be released from the lipoproteins in lysosomes after the lipoproteins have been taken up by the cells. In addition, macrophages are capable of degrading aggregated LDL extracellularly, in structures called extracellular synapses, which leads to the release of unsaturated fatty acids in the close proximity of macrophage plasma membrane.^56^ ATP induces a rapid NLRP3 inflammasome activation, taking full effect within 45 min. The finding that oxLDL inhibits the NLRP3 inflammasome activation in monocytes when added 3 h prior to the activation of the cells with ATP, but not when added simultaneously with ATP (Figure 5b), further supports the hypothesis that oxLDL particles have to be processed for the inhibitory effect. Cell response to activation is a complex combination of numerous individual pathways as well as their crosstalk; therefore, it is plausible that accumulation of oxidised lipids in macrophages can inhibit inflammatory processes via tolerogenicity. Tolerogenicity could be induced by macrophage polarisation.^57^ Thus, if fatty acids direct macrophages towards the anti‐inflammatory M2 phenotype, their metabolism no longer support vigorous responses to proinflammatory stimuli, such as LPS and SAA.^57^

The significance of the inhibitory effect of oxLDL was studied also *in vivo* utilising a mouse model of SAA‐induced peritoneal inflammation. Compared to vehicle‐treated mice, those receiving intraperitoneal injection of oxLDL before the injection of SAA showed significantly milder signs of inflammation, including reduced influx of leukocytes into the peritoneal cavity and a decrease in IL‐1β secretion (Figure 8). These findings suggest that oxLDL can inhibit the SAA‐induced inflammasome activation *in vivo*. Accordingly, we can surmise that the interaction of SAA with oxidised lipoproteins may have significance also in more complex environments *in vivo*, such as the atherosclerotic arterial wall.

## Conclusions

To conclude, both native and oxidised HDL_3_ and LDL significantly inhibited the ability of SAA to induce the secretion of major proinflammatory cytokines from cultured human macrophages. The inhibitory effects of oxidised HDL_3_ and LDL were even more pronounced than those of native HDL_3_ and LDL, which likely inhibit SAA by merely preventing it from interacting with its cell surface receptors. Notably, the oxidised lipoproteins, as well as oxidised lipids extracted from oxidised LDL, inhibited the assembly and activation of the NLRP3 inflammasome, the associated secretion of autophagosomal vesicles and the concomitant secretion of mature IL‐1β. Oxidised LDL inhibited the SAA‐induced inflammation also *in vivo*, suggesting that these modified lipoprotein particles relevant in atherogenesis may act as regulatory agents of the inflammasome activity in inflamed tissues, notably in atherosclerotic lesions.

## Methods

### Materials

Recombinant human SAA was purchased from PeproTech EC Ltd (Rocky Hill, NJ, USA). Its amino acid sequence corresponds to the sequence of SAA 1α isotype except for the addition of Met at the N‐terminus, substitution of Asp for Asn at position 60 and substitution of His to Arg at position 71. According to the manufacturer, the endotoxin level of the product is < 1 EU μg^−1^ protein. Cytochalasin D was from Sigma‐Aldrich (Saint Louis, MO, USA) and the plasma‐derived SAA‐rich acute‐phase HDL (AP‐HDL) from Calbiochem/Merck Biosciences (Darmstadt, Germany). Dulbecco’s modified Eagle’s medium (DMEM, Walkersville, MD, USA) and Roswell Park Memorial Institute (RPMI) 1640 medium (Verviers, Belgium) were from Biowhittaker/Lonza, penicillin‐streptomycin and L‐glutamine both from Gibco (Grand Island, NY, USA). Serum‐free macrophage medium (SFMM) was purchased from Gibco/Invitrogen (Grand Island, NY, USA), and granulocyte‐macrophage colony‐stimulating factor (GM‐SCF) from Nordic Biosite (Täby, Sweden). ATP and nigericin were from Sigma (Saint Louis, MO, USA). ATP was dissolved in culture media and neutralised with NaOH. Monosodium urate crystals (MSU) were prepared from uric acid (Sigma) as previously described^58^ and stored at −20°C. Their endotoxin content was measured by Pyrogent gel clot assay (Lonza, Saint Louis, MO, USA) and was found to be below the detection limit (< 0.03 EU mL^−1^). 3‐methyl adenine (3‐MA, Calbiochem, Saint Louis, MO, USA) and *N*‐acetyl l‐cysteine (NAC, Sigma) were freshly dissolved in culture media. Tin mesoporphyrin (SnMP, Santa Cruz, Dallas, TX, USA) was dissolved in 0.1 m NaOH, neutralised and sterile filtered. MitoTempo and peroxisome proliferator‐activated receptor (PPAR) antagonists T0070907 and GW6471, all from Sigma, were dissolved in DMSO.

### Isolation and oxidation of lipoproteins

Human LDL (*d* = 1.019–1.050 g mL^−1^) and HDL_3_ (1.125–1.210 g mL^−1^) were isolated from the plasma of fasting normolipidaemic volunteers by sequential ultracentrifugation as described.^59^ Protein concentration in the preparations was measured by the Lowry method using bovine serum albumin as a standard.^60^ The amounts of the lipoproteins in this study are expressed in terms of their protein concentration. For oxidation, lipoproteins were first dialysed overnight at +4°C against PBS and then incubated in the presence of 10 μm CuSO_4_ at +37°C for > 18 h. The oxidation was terminated by adding EDTA to the final concentration of 100 μm. Alternatively, the dialysed LDL was incubated in cigarette smoke‐treated PBS^61^ at +37°C for > 18 h and re‐dialysed before using it in experiments. The degree of oxidation in all cases was determined by thiobarbituric acid reactive substances (TBARS) analysis.

### Formation of SAA‐lipoprotein complexes

LDL‐SAA complex used in the uptake experiments was prepared by mixing LDL and SAA at a molar ratio of 1:1 in PBS and incubating the mixture for 2 h at +37°C. At this ratio, all SAA was bound to LDL, as verified by gel filtration (Superose™6, GE Healthcare, Darmstadt, Germany). HDL_3_‐SAA and LDL‐SAA complexes used in the IL‐1β secretion experiments were prepared by mixing HDL_3_/LDL and SAA to their final experiment concentrations in cell culture medium and incubating the mixture for 1 h at +37°C prior to the experiments.

### Preparation of microemulsion particles

Lipids were extracted from 5 mg LDL or oxLDL as per Folch *et al*. and then evaporated under N_2_.^62^ Microemulsion particles were prepared essentially as described by Ginsburg *et al*.^63^ Shortly, dried lipids were resuspended in 1.5 mL of PBS and sonicated at 50°C for 6 × 5 min using Branson Sonifier^®^ 250 ultrasonic cell homogeniser. The emulsion was centrifuged at 14 000 *g* for 5 min, after which the supernatant was collected and sterile‐filtered for cell culture experiments. The amount of total cholesterol in the microemulsion particles was determined using a commercial kit (Roche, Mannheim, Germany).

### Cell culture

Human primary mononuclear cells (PBMC) were isolated from buffy coats (obtained from the Finnish Red Cross Blood Transfusion Center, Helsinki, Finland) by centrifugation in Ficoll‐Paque gradient as previously described.^64^ Washed cells were suspended in DMEM and seeded on 24‐well plates (1.5 × 10^6^ cells well^−1^). After 1 h, non‐adherent cells were removed by washing the cells 2x with PBS and the medium was replaced with SFMM supplemented with 10 ng mL^−1^ GM‐CSF, 100 U mL^−1^ penicillin and 100 μg mL^−1^ streptomycin. The media were then replaced every 48 h until the differentiated macrophages were used for experiments after 6–7 days of culture. For caspase‐1 activity measurement in peripheral blood monocytes, the PBMCs were let to adhere and the non‐adherent cells were removed by washes. The attached cells were placed in SFMM, after which the cells were used for experiments.

Human monocytic leukaemia cell line THP‐1 was purchased from American Type Culture Collection. The cells were maintained in RPMI‐1640 supplemented with 2 mm GlutaMax (Gibco, Paisley, UK), 10% FCS (Gibco, Paisley, UK), 25 mm HEPES (Lonza, Walkersville, MD, USA), 100 U mL^−1^ penicillin and 100 μg mL^−1^ streptomycin. Monocyte‐to‐macrophage differentiation was induced by 100 nm phorbol myristate acetate (PMA) (Calbiochem/Merk, Billerica, MA, USA) for 72 h. HEK‐blue TLR4 reporter cells (InvivoGen, cat. hkb‐htlr4, San Diego, CA, USA) were cultured in DMEM, containing 10% foetal bovine serum and 100 U mL^−1^ penicillin, and 100 μg mL^−1^ streptomycin.

### Ethics statement

Buffy coats were obtained from healthy blood donors, who had signed an informed consent document. The buffy coats were by‐products from the preparation of blood products for clinical use. The use of buffy coats in monocyte isolation was approved by the Finnish Red Cross Blood Service.

### Cell stimulations

Human primary macrophages and PMA‐differentiated THP‐1 cells, called THP‐1 macrophages, were incubated for 5–18 h at +37°C in fresh SFMM or RPMI, respectively, containing SAA (3 μg mL^−1^) or AP‐HDL (corresponding to 10 μg mL^−1^ SAA). Alternatively, macrophages were pre‐incubated with HDL_3_, oxHDL_3_, LDL or oxLDL (10 ‐ 400 μg mL^−1^), or with LDL or oxLDL‐derived microemulsion particles (450 μg mL^−1^ cholesterol) for 1 h, after which SAA (3 μg mL^−1^) was introduced either by adding it as such into the culture media or by washing the cells first and adding SAA into the fresh culture media. In experiments with oxidised lipoproteins, same volume of 100 μm EDTA‐PBS without lipoproteins was used as mock treatment. The experiment was also conducted in reverse order, or in the presence of cytochalasin D (1 μg mL^−1^), which was added 1 h before other stimulants. For activation of the NLRP3 inflammasome with activators other than SAA, primary macrophages were primed with LPS (1 µg mL^−1^) for 3 h after which oxLDL was added for 1 h, followed by NLRP3 inflammasome activation with ATP (5 mm, 30 min), nigericin (4 μm, 1 h) or MSU crystals (200 µg, 4 h). THP‐1 macrophages were subjected to oxHDL_3_ or oxLDL (200 μg mL^−1^), and after a 1‐h incubation, nigericin (4 μm) was added and incubation was continued for an hour. For activation of noncanonical inflammasome, THP‐1 macrophages were preincubated in the presence of LDL or oxLDL (200 μg mL^−1^) for 1 h and then transfected with ultrapure LPS (InvivoGen, LPS‐EB, E. coli 0111:B4) for 6 h, using Lipofectamine 2000 (5 µl mL^−1^, Invitrogen, Carlsbad, CA, USA) according to manufacturer’s instructions. For AIM2 inflammasome activation, THP‐1 macrophages were differentiated with 50 nm PMA for 48 h, after which they were preincubated in the presence of LDL or oxLDL (200 μg mL^−1^) for 1 h and then transfected with poly(dA:dT) (InvivoGen) for 5 h. For inhibition assays, THP‐1 macrophages were pretreated with SnMP (5 mm) for 3 h, after which oxLDL (200 µg mL^−1^) was added and the incubation was continued for 1 h. After this, cells were challenged with SAA 3 mg mL^−1^ and the incubation was continued for 5 h. THP‐1 macrophages were pretreated with 3‐MA (5 mm) together with oxLDL (100 or 400 µg mL^−1^) for 1 h and activated with SAA 3 mg mL^−1^ for 5 h. Primary monocytes were activated for 4 h 45 min with LPS, during which oxLDL was added for 3 h 45 min or simultaneously with ATP, and finally, monocytes were activated with ATP for the last 45 min of the incubation.

### Analysis of TLR4 activation

HEK‐blue TLR4 reporter cells were used to study the activation of human TLR4. The levels of secreted embryonic alkaline phosphatase (SEAP) reflect the TLR4‐induced activation of NF‐κB and AP‐1. The cells were cultured per manufacturer’s recommendations, except that the cells were seeded to wells one day before the experiment and the experiments were carried out in SFMM. The HEK‐blue TLR4 cells were changed into SFMM before addition of lipoproteins (no wash group) or after a 1‐h lipoprotein activation and before SAA was applied, to remove the lipoproteins (wash group). The supernatants were collected 24 h after addition of lipoproteins, and SEAP activity was detected in the supernatants by QUANTI‐Blue reagent (InvivoGen), according to manufacturer’s recommendations.

### Animals and induction of peritoneal inflammation

Experiments were conducted in conformity with the Finnish regulations, and the protocols were approved by The National Animal Experiment Board. Wild‐type female C57BL/6J mice, aged 8‐13 weeks, were purchased from Harlan Laboratories (Venray, Netherlands). The mice were housed (5 per cage) in conditions controlled for light/dark cycle, temperature and humidity, and they had access to a standard chow‐diet (Teklad Global 16% protein Rodent diet, Harlan Laboratories) and water ad libitum. Mice received buprenorphine (0.1 μg g^−1^) subcutaneously as analgesic 1 h before dosing the experimental substances. First, mice were given intraperitoneal (i.p.) injection of oxLDL (400 μg 300 μL^−1^ per mouse) or vehicle (PBS, 300 μL per mouse), followed by an i.p. injection of SAA (30 μg 300 μL^−1^ per mouse) or vehicle (PBS, 300 μL per mouse) 1 h later. Peritoneal inflammation was allowed to develop for 4 h, after which the mice were terminally anesthetised by isoflurane and the peritoneal cavity was lavaged with 2 mL of 0.1% BSA in PBS. The peritoneal fluid was collected and centrifuged at 200 *g* for 5 min, after which the cells were counted with NucleoCounter NC‐200™ (Chemometec) and the supernatant was analysed for IL‐1β concentration.

### Cytokine and LDH analyses

Human IL‐1β and TNF concentrations in the macrophage culture media or mouse IL‐1β concentration in the peritoneal fluid were analysed using commercial enzyme‐linked immunosorbent assays (ELISA) according to the manufacturers’ protocols (R&D Systems, Minneapolis, MN, USA). Pyroptotic cell death was assessed by analysing the lactate dehydrogenase (LDH) with the Cell death detection kit (Roche) according to manufacturer´s recommendations.

### Lipid extraction analysis by thin layer chromatography

Cellular lipids were extracted from the macrophages with hexane‐isopropanol (3:2 v/v). The solvent was evaporated, lipids were redissolved in chloroform‐methanol (2:1 v/v), and the samples were applied onto silica‐coated thin layer chromatography (TLC) plates (CAMAG) using an automatic TLC sampler (Sampler3; CAMAG). Hexane/diethyl ether/concentrated acetic acid/H_2_O (130:30:2:0.5 v/v) was used as the mobile phase for the analysis. The lipids were visualised by dipping the TLC plate into 3% CuSO_4_ / 8% H_2_PO_4_ and by heating the plate at 150°C for 10 min. The lipids were detected with an automatic TLC plate scanner (Scanner 3, CAMAG) and analysed using TLC Evaluation Software (CAMAG). After the lipid extraction, the cells were lysed in 0.2 m NaOH and their protein content was determined by the Lowry method.

### Quantitative real‐time RT‐PCR analysis

Stimulated primary macrophages were harvested, and their total cellular RNA was purified using the RNeasy kit (Qiagen, Hilden, Germany). The RNA was converted to cDNA using Moloney murine leukaemia virus reverse transcriptase and random hexamers (Promega, Madison, WI, USA) or iScript cDNA synthesis kit (Bio‐Rad, Hercules, CA, USA). For quantitative real‐time RT‐PCR, the cDNA was amplified in duplicates using TaqMan^®^ Universal PCR Master Mix (for *IL1B*, *TNF* and *GAPDH*) with gene‐specific primers and fluorogenic TaqMan probes when applicable on an ABI PRISM 7500 sequence detector system (Applied Biosystems, Warrington, UK). The data were normalised relative to *GAPDH*. The expression of *NFKBIA*, *CXCL10*, *IRF1*, *IL‐6*, *CCL2* and *GAPDH* was analysed using IQ SYBR Green Supermix (Bio‐Rad), and PCR was performed in the iQ5 real‐time qPCR detection system (Bio‐Rad). The data were normalised relative to *GAPDH,* and the relative units were calculated using the comparative Ct method. The used oligonucleotide sequences are found in Supplementary table 1.

### Immunoblot and silver stain analysis

For Western blotting and silver staining, THP‐1 macrophages were pretreated with oxLDL 200 µg mL^−1^ for 1 h and further challenged with SAA (3 µg mL^−1^) for 5 h. Equal volumes of cell culture media were concentrated using Amicon ultra centrifugal concentrators with a 10 kDa cut‐off (Millipore, Cork, Ireland). Intracellular proteins were blotted from THP‐1 macrophages lysed in 1x cell lysis buffer (Cell Signaling Technology, Danvers, MA USA), supplemented with protease inhibitor cocktail (Roche) and phosphatase inhibitors (Pierce, Carlsbad, CA, USA). Proteins were resolved by SDS‐PAGE (TGX Stain‐free fast cast, Bio‐Rad) and transferred on PVDF membrane. Membranes were incubated o/n at +4°C with antibodies raised against mature human IL‐1β (Cell Signaling, 12242S), ASC (Adipogen, AL177), proform of IL‐1β (sc‐7884), Annexin‐1 (sc‐12740) and Alix (sc‐53540) (all from Santa Cruz), CD11c, also known as integrin alpha X (ITGAX) (EP1347Y) and SQSTM1 (ab56416) (both from Abcam), caspase‐1 (AG‐20B‐100) and NLRP3 (AG‐20B‐0014) (both from Adipogen), and LC3 (Novus Biologicals, NB100‐2331). Antibodies were detected with HRP‐conjugated anti‐mouse (P0447) or anti‐rabbit antibodies (P0448) (Dako). Equal loading of secreted proteins was verified by loading equal volumes of the equalised media concentrates in each lane. The intensities of intracellular proteins were normalised to the intensity of total protein loading on each lane using the Bio‐Rad Stain‐free™ technology^65^ and a Chemidoc MP imaging system (Bio‐Rad). Total protein staining was detected with commercial polyacrylamide gels (Bio‐Rad), on which tryptophan residues contained in the loaded proteins are modified so that they can be visualised from PVDF membranes.

### ASC immunofluorescence staining

ASC speck detection was performed as previously described, with minor modifications.^66^ Briefly, THP‐1 macrophages were incubated in the presence of oxLDL (400 µg mL^−1^) for 1 h and then activated with SAA (3 µg mL^−1^) for 5 h. PFA‐fixed cells were permeabilised with 0.3% Triton X‐100 and stained with ASC antibody (MBL, D086‐3). Goat anti‐mouse IgG1 (Alexa, Invitrogen, A21121) was used as secondary antibody, and DAPI was used to stain the nuclei. The speck formation was analysed by epifluorescence microscopy (Leica DM6000B). The number of specks per field was divided by total number of cells per field, and the speck formation was expressed as percentages. In each of the four experiments, 10 fields (80 cells per field on average) were counted for each treatment.

### Caspase‐Glo assay

Active caspase‐1 was detected in the culture media and in the cell lysates by Caspase‐Glo 1 Inflammasome Assay (Promega) according to manufacturers’ protocols. Luminescence was measured by a luminometer (Tecan SW Sparkctl. Magellan V2.2).

### Statistical analysis

Calculations were performed using GraphPad Prism vs. 8.0 software. Overall significance level between the stimulated and control groups or between stimulated and inhibited groups was analysed using one‐way ANOVA, followed by the Sidak, Dunnett and Tukey‐Kramer multiple comparisons tests when appropriate. Statistical significance was set at *P* < 0.05. The data are shown as mean ± SEM.

## Conflict of interest

The authors declare no conflicts of interest.

## Author contributions

**Katariina Nurmi:** Conceptualization; Data curation; Formal analysis; Funding acquisition; Investigation; Methodology; Project administration; Validation; Visualization; Writing‐original draft; Writing‐review & editing. **Katri Niemi:** Conceptualization; Data curation; Formal analysis; Investigation; Methodology; Writing‐original draft; Writing‐review & editing. **Ilona Kareinen:** Conceptualization; Investigation; Methodology; Writing‐review & editing. **Kristiina Silventoinen:** Investigation; Visualization; Writing‐review & editing. **Martina Lorey:** Investigation; Methodology; Writing‐review & editing. **Yan Chen:** Investigation; Writing‐review & editing. **Vesa‐Petteri Kouri:** Conceptualization; Formal analysis; Investigation; Methodology; Writing‐review & editing. **Jukka Parantainen:** Investigation; Writing‐review & editing. **Timo Juutilainen:** Conceptualization; Resources; Writing‐review & editing. **Katariina Öörni:** Conceptualization; Methodology; Resources; Writing‐review & editing. **Petri Kovanen:** Conceptualization; Resources; Writing‐review & editing. **Dan Nordström:** Conceptualization; Funding acquisition; Project administration; Resources; Writing‐review & editing. **Sampsa Matikainen:** Conceptualization; Funding acquisition; Project administration; Resources; Writing‐review & editing. **Kari Eklund:** Conceptualization; Formal analysis; Methodology; Project administration; Resources; Software; Supervision; Validation; Writing‐review & editing.
