# Supplementary material for: Native and oxidised lipoproteins negatively regulate the serum amyloid A‐induced NLRP3 inflammasome activation in human macrophages
Source: Clin Transl Immunology. 2021 Aug 3;10(8):e1323. doi: 10.1002/cti2.1323 (PMC8329955; doi:10.1002/cti2.1323)
Supplement: Supplementary file 1 [file CTI2-10-e1323-s001.pdf]

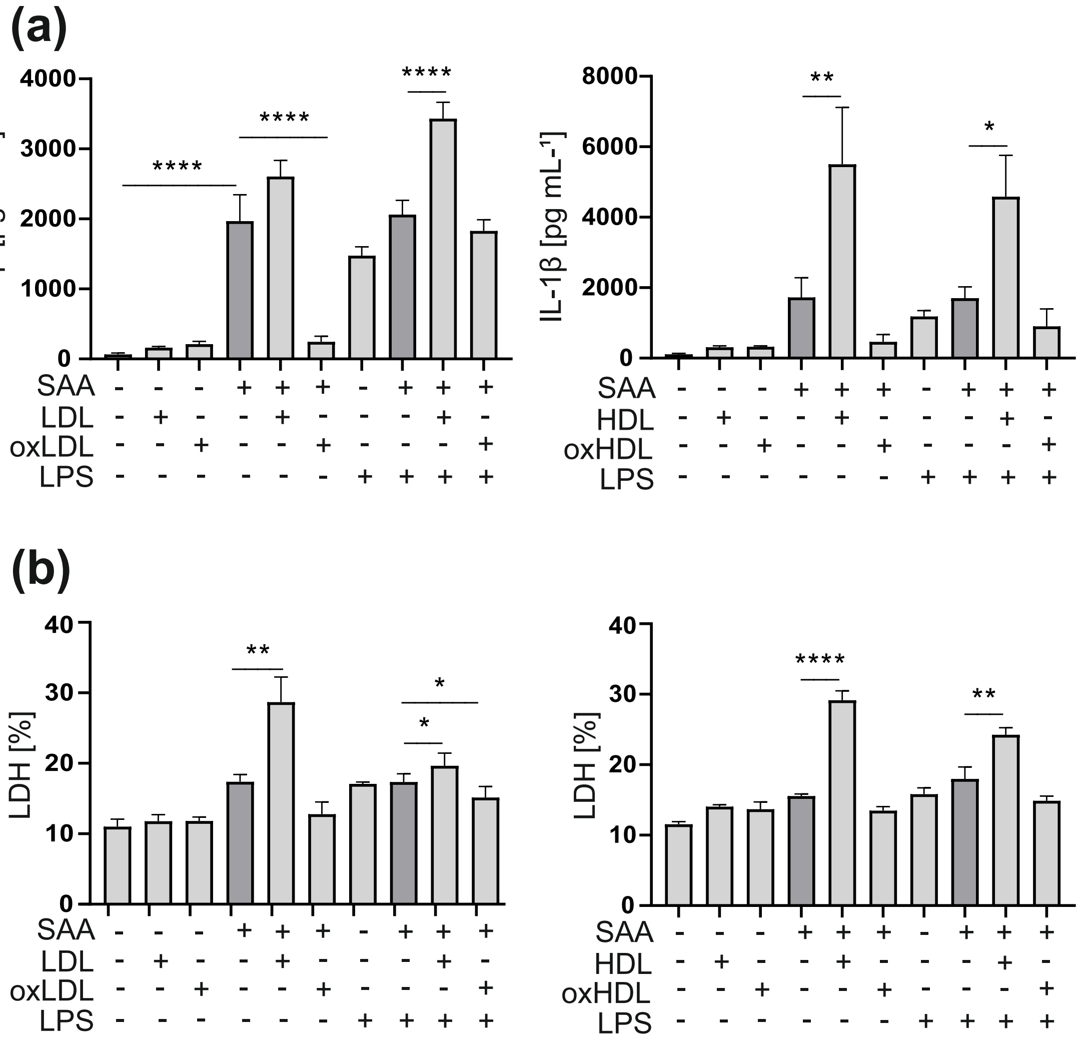

Supplementary Figure 1

THP-1 macrophages were primed with LPS (Sigma L3012, 1 μg mL^{-1}, 3 h), after which they were subjected to native or oxidised LDL or native or oxidised HDL_3 (200 μg mL^{-1}) for 1 h, and then to SAA (3 μg mL^{-1}) for 5 h. **(a)** Secretion of IL-1β was analysed from cell culture media by ELISA. Native and oxidised LDL, left panel; native and oxidised HDL_3, right panel. **(b)** LDH was analysed from cell culture media. Native and oxidised LDL, left panel; native and oxidised HDL_3, right panel. The mean values shown are from 4 experiments. **P*<0.05, ***P*<0.01, *****P*<0.0001.

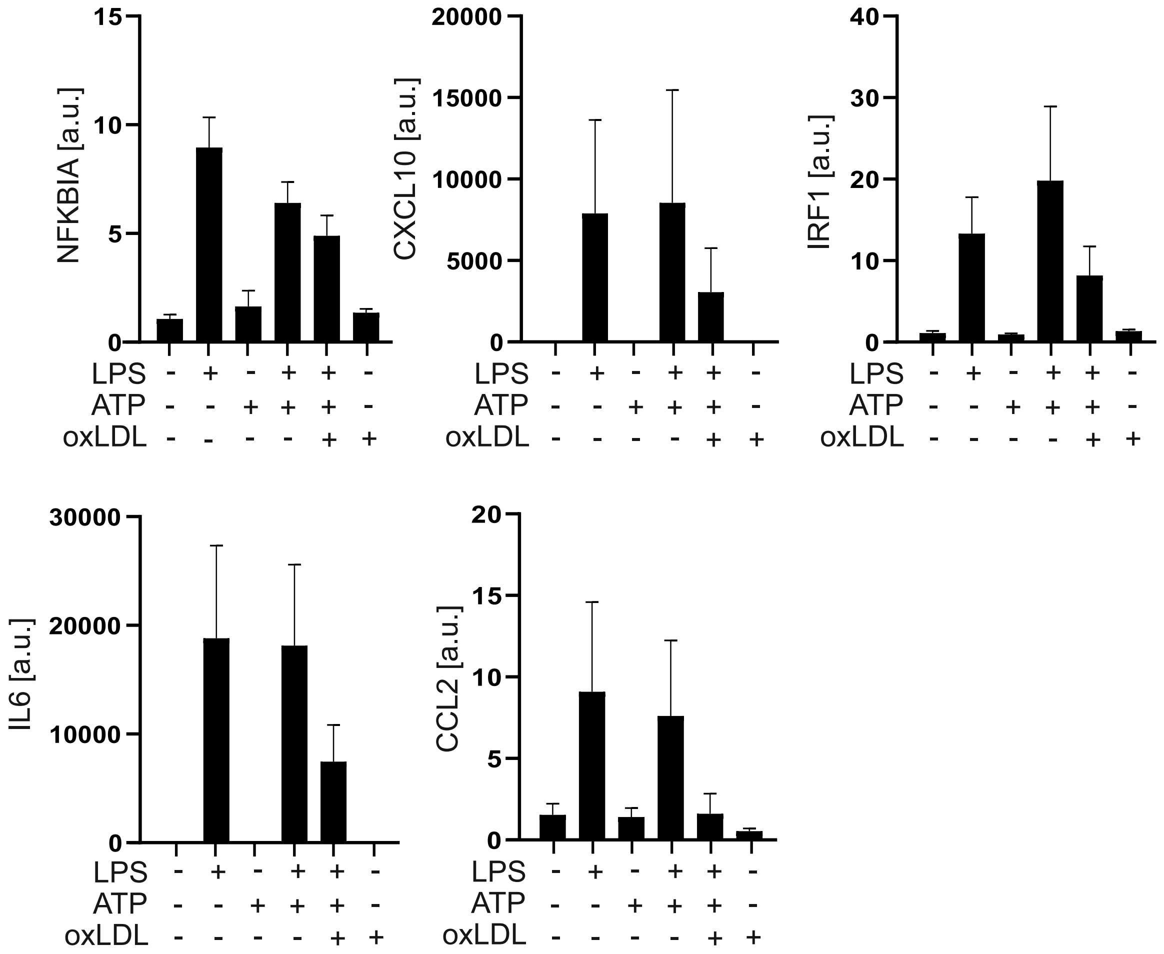

Supplementary Figure 2

Human primary macrophages were primed with LPS for 3 h, then subjected to oxidised LDL 400 µg mL^{-1} for 3 h and activated with ATP. The expression of *NFKBIA*, *CXCL10*, *IRF1*, *IL6* and *CCL2* mRNA was analysed by quantitative real-time RT-PCR and is expressed as arbitrary units (a.u.). The mean values shown are from 4 experiments.

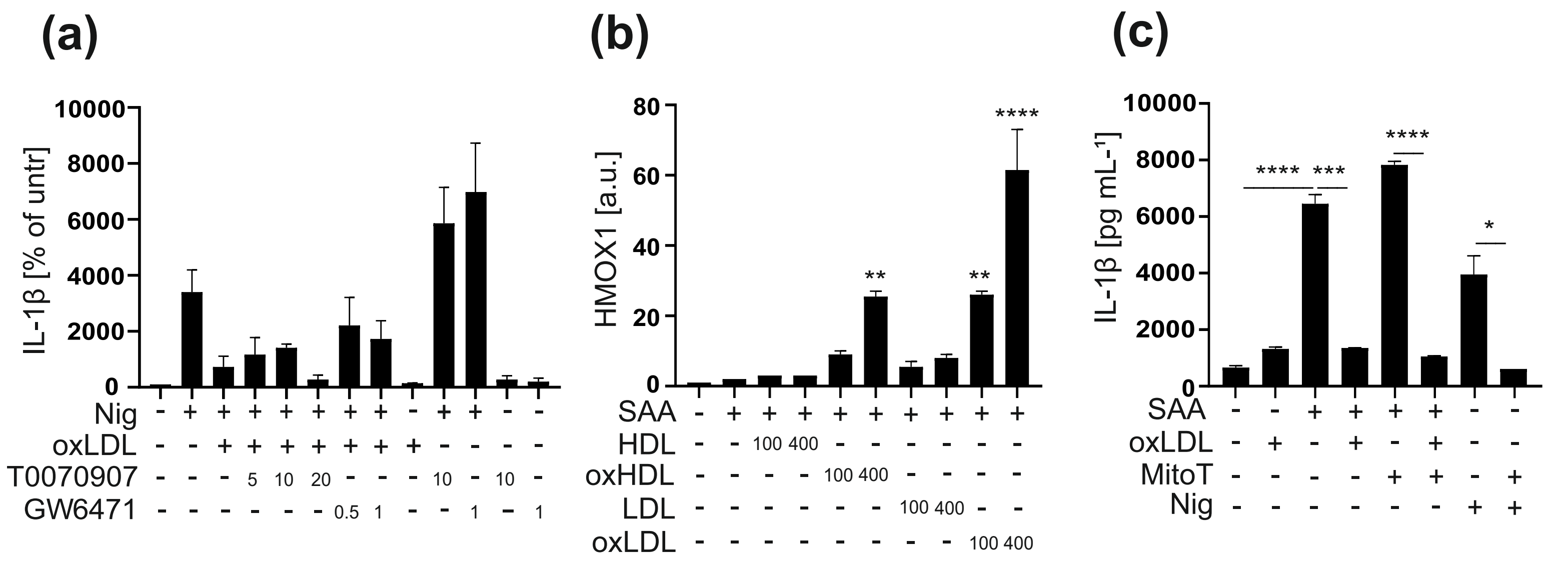

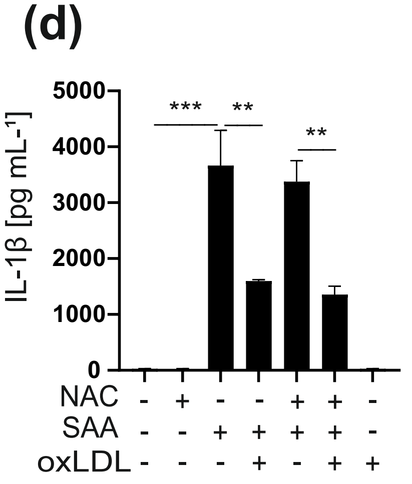

Supplementary Figure 3

(a) THP-1 macrophages were treated with PPAR antagonists T0070907 and GW6471 in the indicated concentrations (µM) for 1.5 h, then oxidised LDL 400 µg mL^{-1} was added for 3 h, after which the cells were activated with nigericin for 1 h. Cell culture media were analysed for IL-1β concentrations by ELISA. The data are expressed relative to untreated cells. **(b)** Human primary macrophages were subjected to native and oxidised HDL_3 and LDL at indicated concentrations for 1 h and then to SAA (3 µg mL^{-1}) for 5 h. After the incubation, the cells were harvested and analysed for *HMOX1* mRNA levels by quantitative real-time RT-PCR. Data are expressed as arbitrary units (a.u.). **(c)** THP-1 macrophages were pretreated with MitoTEMPO 300 µM for 1 h, after which oxidised LDL 400 µg mL^{-1} was added for 1 h, and then the cells were activated with SAA 3 µg mL^{-1} for 5 h. Cell culture media were analysed for IL-1β concentrations by ELISA. **(d)** THP-1 macrophages were pretreated with N-acetyl cysteine (NAC) 0.5 µM for 1 h, after which oxidised LDL 200 µg mL^{-1} was added for 1 h, and finally the cells were activated with SAA 3 mg mL^{-1} for 18 h. Cell culture media were analysed for IL-1β concentrations by ELISA. The mean values shown are from 2 experiments. **P <0.01, ***P <0.001, ****P <0.0001.

Supplementary table 1. Primers

**Gene**	**F** (5'-3')	**R** (5'-3')	**probe**
IL1B	TTACAGTGGCAATGAGGATGAC	GTCGGAGATTCGTAGCTGGAT	FAM-AACAGATGAAGTGCTCCTTCCAGGACC- BHQ1
TNF	GCTGCACTTTGGAGTGATCG	GTTTGCTACAACATGGGCTACAG	FAM- CCCAGGCAGTCAGATCATCTTCTCGA-BHQ1
GAPDH	CCACATCGCTCAGACACCAT	GGCAACAATATCCACTTTACCAGAG	FAM-CCAATACGACCAAATCCGTTGACTCC-BHQ1
NFKBIA	CGGACTGCCCTTCACCTCGC	GTATCCGGGTGCTTGGGCGG	
CXCL10	GCAAGCCAATTTTGTCCACGTGTTG	CAGCCTCTGTGTGGTCCATCCTT	
IRF1	CATGAGACCCTGGCTAGAGATG	TCCGGAACAAACAGGCATCC	
IL6	AGGAGACTTGCCTGGTGAAA	GAGGTGCCCATGCTACATTT	
CCL2	ATTCCCCAAGGGCTCGCTCA	GGTTTGCTTGTCCAGGTGGTCC	
GAPDH	TCACCATCTTCCAGGAGCGA	TGGACTCCACGACGTACTCA	
